# Case report: Moderate therapeutic response to Bevacizumab in late-onset Labrune syndrome

**DOI:** 10.3389/fneur.2022.968403

**Published:** 2022-09-27

**Authors:** Meiping Wang, Jinmei Lu, Xiaoxi Wang, Xiaoqun Ba, Dengchang Wu, Jianfang Zhang, Jiajia Zhou, Kang Wang

**Affiliations:** ^1^Department of Neurology, First Affiliated Hospital, School of Medicine, Zhejiang University, Hangzhou, China; ^2^Department of Pathology, First Affiliated Hospital, School of Medicine, Zhejiang University, Hangzhou, China

**Keywords:** Labrune syndrome, treatment, Bevacizumab, epilepsy, late-onset

## Abstract

Labrune syndrome (LS) is caused by *SNORD118* gene mutations with a particular neuroimaging of white matter disease, intracranial calcification, and cysts. There was no effective treatment until now. An 18-year-old man with infancy-onset LS was first treated with vascular endothelial growth factor (VEGF) inhibitor Bevacizumab for 1 year, resulting in significant clinical and radiological improvements. We adopted a similar regimen in a patient with late-onset LS and demonstrated moderate cognitive improvements but without changes in imaging. As such, Bevacizumab could potentially be clinically effective in adult-onset LS with great safety.

## Introduction

Labrune syndrome (LS), also known as leukoencephalopathy with cerebral calcifications and cysts (LCC), is characterized by a neuro-radiological triad of white matter disease, intracranial calcification, and cysts (1). LS is a rare autosomal recessive disorder, caused by pathogenic variants in *SNORD118* (2). The age of clinical onset is variable and ranges from infancy, childhood, and early adulthood to late adulthood. The clinical manifestations of LS are diverse, including seizures, motor deficit, and cognitive impairment, among others. Current treatments for LS are very limited. Fay and team (3) first reported an 18-year-old man with infancy-onset LS receiving administration of Bevacizumab, a monoclonal anti-vascular endothelial growth factor (VEGF) antibody for 1 year, and subsequently showed encouraging outcomes both clinically and radiologically, shining a light on this devastating disease. Therefore, we adopted a similar treatment regimen in an adult patient with onset LS and observed its efficacy and safety during and after treatment.

## Materials and methods

**Figure 1:**
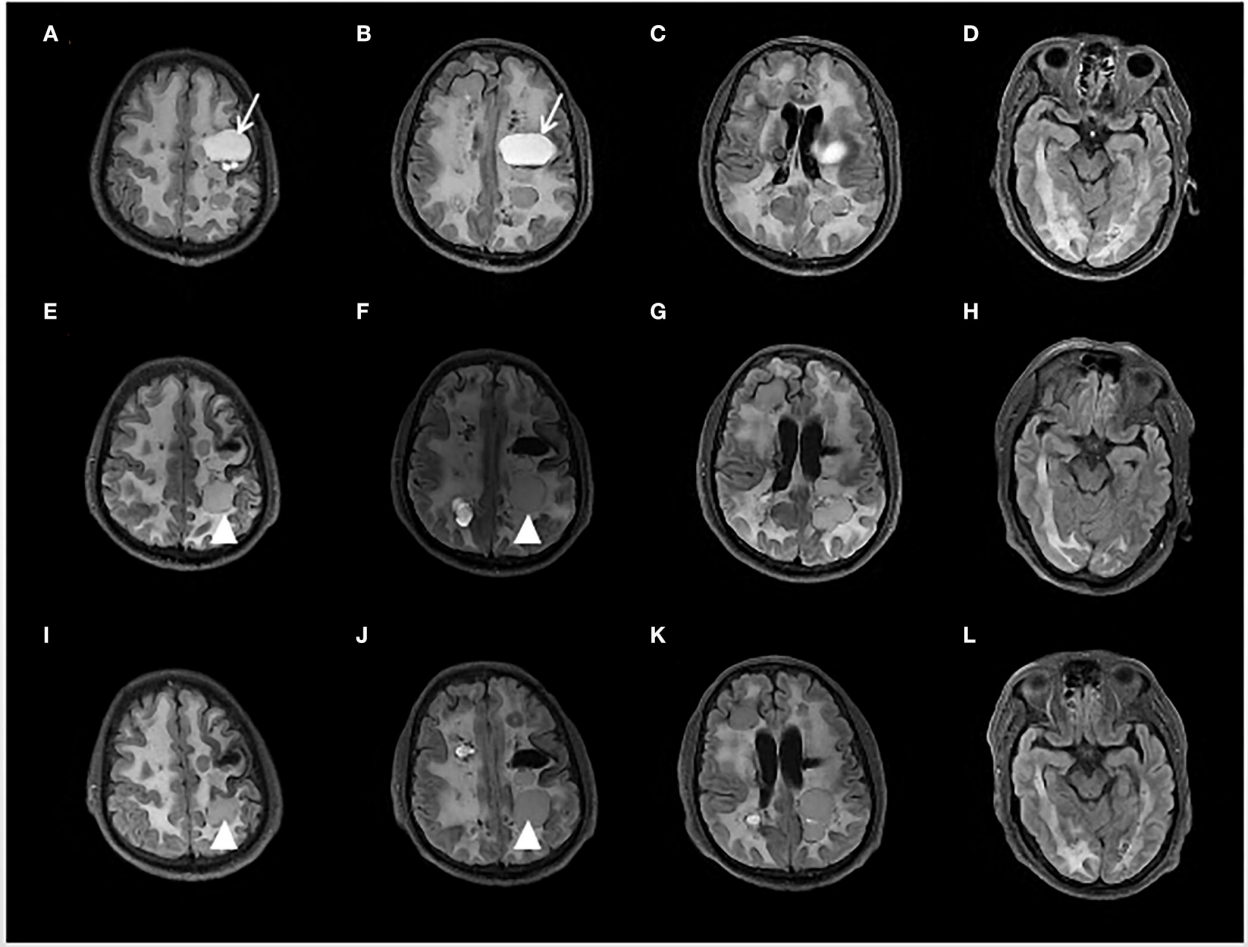
Brain fluid-attenuated inversion recovery (FLAIR) sequence of initial presentation **(A–D)**, 6 months **(E–H)**, and 12 months **(I–L)** after Bevacizumab treatment. The initial MRI showed multiple cystic foci and white matter lesions in bilateral cerebral hemispheres. The left mass cystic foci [showed as an arrow in **(A,B)**] was decompressed by an Ommaya reservoir. After 6 months treatment of Bevacizumab, another left frontal lobe cystic foci [showed as an arrowhead in **(E,F,I,J)**] was slightly enlarged.

A 53-year-old man had his first episode of generalized tonic-clonic seizure in November 2017. Two months later, he developed jerks restricted to the right limb followed by transient weakness. At a local hospital, a brain MRI showed diffuse hyperintensity in periventricular and deep white matter, and numerous cystic lesions in different sizes in bilateral hemispheres with occupying effects were observed in some images (see Figure 1). The susceptibility-weighted imaging (SWI) and CT indicated wide-spreading remote bleeding and calcification. He was given sodium valproate 0.5 g twice a day and levetiracetam 0.5 g twice a day, and seizure frequency stabilized to 2–4 times each year. Due to progressive cognition decline and persistent weakness of the right limb, he was admitted for diagnostic evaluation and management in October 2020.

**Figure 2:**
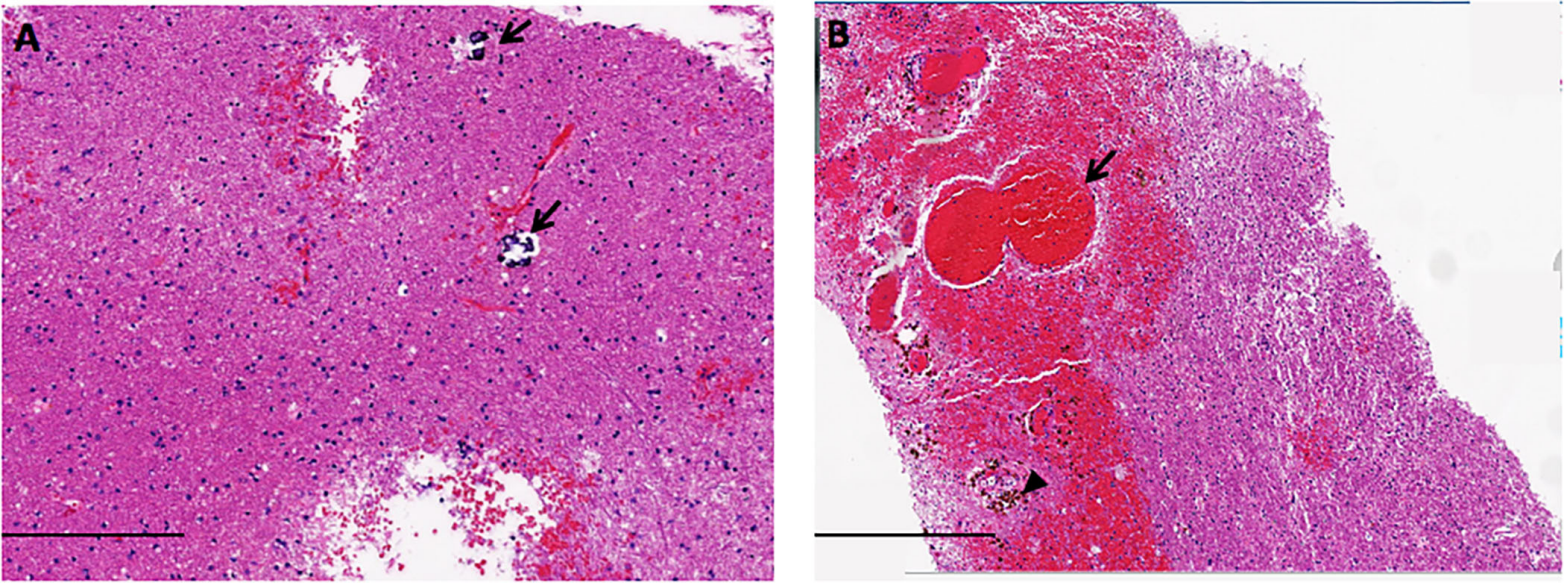
The neuropathology of LS showed small calcification scattered [arrow in **(A)**], atypical gliosis, hemorrhage [arrow in **(B)**], and hemosiderin deposition [arrowhead in **(B)**], in the white matter (scale bars A = 200 μm, B = 400 μm).

Physical examination on admission exhibited decreased muscle strength in right limbs for grade 2/5 with the presence of the Babinski sign. Mini-Mental State Examination (MMSE) scored 7 points, and Modified Rankin Scale (mRS) graded 5. Extensive diagnostic tests were unremarkable excluding underlying infections. The ophthalmological investigation was normal. Biopsy of the right frontal lobe showed atypical gliosis, clustered venule hyperplasia, hemosiderin deposition, and small calcification scattered within white matter, consistent with LS (see Figure 2). The genetic testing disclosed compound heterozygous variants (*n.3C*>*T and n.24C*>*T*) of the patient in the *SNORD118* gene, and both of them were previously reported as pathogenic, confirming the diagnosis of LS.

The existing anti-seizure medication (ASM) regimen was continued where an Ommaya reservoir was placed into a 4 × 2.5 cm cyst in the left frontal lobe to decompress involved structures. After the procedure, the muscle strength of his right limb improved to grade 4/5 in 1 week, but with no change in MMSE performance.

After obtaining informed consent from the patient's wife and approval from the First Hospital of Zhejiang Province's ethics committee, he was treated with Bevacizumab 350 mg (5 mg/kg) intravenously biweekly for 12 months. He was required to have three assessments at the 3^rd^, 6^th^, and 12^th^ months during the treatment, and one additional evaluation at the 6 th-month post completion of the treatment. The assessments included MMSE, seizure inquiry, brain MRI, observation for any side effects relating to bleeding events, hypertension, urinalysis, and blood workup including blood cell count, liver function, and renal function that were conducted monthly at a local hospital. This study was registered at the Chinese Clinical Trial Registry (No. ChiCTR 2000041205).

## Results

Clinical outcomes in 3^rd^ month after Bevacizumab infusion showed significant cognitive improvement evidenced by MMSE score rising from 7 to 15 points, and it continued to increase to 17 points at 6^th^ follow-up. After that, cognitive function seemed to reach a plateau even after stopping the treatment. His seizures were well-controlled except for only one seizure relapse in the 12^th^ month of the trial presumably due to abrupt discontinuation of ASM, but he remained seizure-free after resuming ASM. Motor deficit of right limbs stabilized at grade 4/5 strength throughout the clinical trial.

From a radiological perspective, compared with the brain MRI before the trial, the MRI scans at the 6^th^ month and at the 12^th^ month showed minimal changes except for slight enlargement in one cyst (see Figure 1).

The patient did not report any side effects during the treatment, and all lab tests on a regular basis came back normal.

## Discussion

Labrune syndrome is caused by the mutation of the *SNORD118* gene encoding the box C/D snoRNA U8, first described by Labrune et al. (1, 2). LS was presented with wide neurological dysfunctions like progressive cognitive impairments, gait disturbance, and seizures but on rare occasions, LS might have systemic involvements such as multiple hepatic and renal cysts (4). Our patient presented with seizures, cognitive impairments, and right hemiparesis. Genetic analysis of our patient confirmed compound heterozygous SNORD118 mutations, n.3C>T and n.24C>T. The site of n.3C>T mutation had been reported in 4 patients with various ages of onset, from 2 years to 30 years (5, 6), indicating that site of genetic mutation did not correlate with the age of presentation. While n.24C>T mutation had been reported in an 11-year-old onset male (5) who presented with symptoms of raised intracranial pressure. Both mutations of n.3C>T and n.24C>T were located at the 5' end of pre-U8, which might be associated with impaired U8 processing (5). Taken together, no evidence showed an obvious genotype-phenotype correlation in LS.

Radiologically, LS is characterized by a combination of leukoencephalopathy, brain calcifications, and cysts. Of note, this neuro-radiological triad is also shared by Coats plus syndrome (CPS) whose disease-causing gene is *CTC1*. Nevertheless, LS is readily differentiated from CPS, as the latter is a multi-system disorder with a tendency to involve various structures other than the brain, such as the retina, bones, and gastrointestinal system. Considering their pathological similarities in the brain, LS and CPS are currently grouped as one under the term cerebroretinal microangiopathy with calcifications and cysts.

No curative treatment is available for LS. The main symptomatic treatments are cystic surgical procedures such as cystic puncture, cystic resection, and cysto-ventriculoperitoneal shunting. Up until 2021, two patients were treated with Ommaya reservoir placement in cyst (7, 8), where both patients achieved marked improvements in motor function. The limb muscle strength of our patient also improved within one week of the placement of the Ommaya reservoir at low risk, indicating that this approach could be prioritized when motor-associated structures such as pyramidal tracts and basal ganglia, were compressed.

Pathologically, LS is characterized by microangiopathy as ectasia of small cerebral vessels and deposition of intermediate filaments called “Rosenthal fiber”. With VEGF being identified as a key regulator in angiogenesis and vascular permeability, Bevacizumab, the first VEGF inhibitor which was initially approved for use in cancer treatment, was subsequently administered *via* injection into the eye for off-label use in age-related macular degeneration, resulting in great benefits. Additionally, Bevacizumab was discovered to profoundly reduce retinal edema and exudates in CPS (9, 10). Inspired by the success in proliferative neovascular eye diseases as well as the common microvascular pathology between LS and CPS, Fay et al. (3) reported that Bevacizumab improved bradykinesia and range of motion in a patient with early-onset LS, and partly reversed cysts and white matter lesions on MR while Martínez-Matilla et al. (11) reported an infant with LS showed apparent radiological improvements with unchanged clinical status after receiving Bevacizumab. By contrast, although our case demonstrated minimal radiological alterations, suggesting irreversibility on imaging for late-onset patients, long-term clinical improvements especially in cognition, even after discontinuation of Bevacizumab, were impressive and encouraging. One limitation of this study is the use of MMSE in cognitive assessment as the patient failed to complete more thorough cognitive evaluations at the beginning of the trial due to poor cooperation. Besides, further studies are needed to confirm the pathology of microangiopathy in late-onset patients. Additionally, Bevacizumab might contribute to his seizure control as seizures in LS were reported to be drug resistant (12, 13). In terms of safety, the patient was well tolerated at a dosage of 5 mg/kg throughout the study and no obvious side effects were observed. Although Bavacizumab appeared to be effective in treating LS, optimal dosage and administration duration were unknown.

In summary, Bevacizumab might be clinically beneficial in late-onset LS with great safety, though marginal changes in imaging. More case studies and longer follow-ups are needed.

## Data availability statement

The datasets presented in this article are not readily available because of ethical and privacy restrictions. Requests to access the datasets should be directed to the corresponding authors.

## Ethics statement

The studies involving human participants were reviewed and approved by First Hospital of Zhejiang Province's Ethics Committee. The patients/participants provided their written informed consent to participate in this study. Written informed consent was obtained from the individual(s) for the publication of any potentially identifiable images or data included in this article.

## Author contributions

MW wrote the first draft of the manuscript and interpreted data. JL and DW collected the clinical data and followed patient. XW and XB confirmed pathology. JZho and JZha collected MRI data. KW designed and registered the study and revised the manuscript. All authors contributed to the article and approved the submitted version.

## Funding

This work was supported by a grant from Zhejiang Province Natural Science Foundation (Grant No. LY19H090020).

## Conflict of interest

The authors declare that the research was conducted in the absence of any commercial or financial relationships that could be construed as a potential conflict of interest.

## Publisher's note

All claims expressed in this article are solely those of the authors and do not necessarily represent those of their affiliated organizations, or those of the publisher, the editors and the reviewers. Any product that may be evaluated in this article, or claim that may be made by its manufacturer, is not guaranteed or endorsed by the publisher.
